# Imaging Mass Cytometry Analysis of immune Checkpoint Inhibitor-Related Pneumonitis: A Case Report

**DOI:** 10.3389/fimmu.2022.899971

**Published:** 2022-07-15

**Authors:** Yuan Cheng, Xiao-Ming Wang, Qin Hu, Kunyan Sun, Xiang Zhao, Meng Zhang, Guangfa Wang, He Wang, Yan Xiong

**Affiliations:** ^1^ Department of Respiratory and Critical Care Medicine, Peking University First Hospital, Beijing, China; ^2^ School of Basic Medical Sciences, Wuhan University, Wuhan, China; ^3^ Faculty of Environment and Life, Beijing University of Technology, Beijing, China; ^4^ Department of Radiology, Peking University First Hospital, Beijing, China; ^5^ Department of Pathology, Peking University First Hospital, Beijing, China

**Keywords:** imaging mass cytometry, immune-related adverse event, immune checkpoint inhibitor-related pneumonitis, memory T cells, heavy-metal tagged antibodies

## Abstract

Immune checkpoint inhibitor-related pneumonitis (CIP) is a rare but well-recognized immune-related adverse event (irAE), causes 35% of irAE related deaths. However, the mechanism of CIP remains unclear and no evidence-based treatment except for glucocorticoids is available. Herein, we report the case of a patient with metastatic bladder cancer who received tislelizumab and was diagnosed with CIP. The patient underwent transbronchial cryobiopsy. The patient was treated with glucocorticoid, but CIP recurred when the glucocorticoid tapering. The paraffine-embedded lung tissue was sectioned, stained with 31 heavy-metal tagged antibodies, and analyzed using imaging mass cytometry (IMC) technology. We identified multiple immune cell subsets in the lung tissue and observed the infiltration of memory T cells and the CD4^+^ DC subset. The data indicated the great potential of IMC technology in the identification and characterization of irAEs. Further investigation is warranted to identify the mechanism of action of CIP.

## Introduction

Since the approval of the first immune checkpoint inhibitor (ICI), ipilimumab, for metastatic melanoma in 2011, ICIs targeting cytotoxic T lymphocyte antigen 4 (CTLA-4), programmed cell death protein 1 (PD-1) and programmed cell death protein ligand 1 (PD-L1) have revolutionized the treatment landscape of numerous cancer types. In the past decade, they have been approved for more than 16 cancer types, including urothelial carcinoma, non-small-cell lung cancer, small-cell lung cancer, melanoma, and renal cell carcinoma (1).

Despite the clinical success of ICI therapy, the incidence of ICI-induced immune-related adverse events (irAEs) has become clinically apparent. The frequency of irAEs varied depending on the cancer type and drug types. Approximately 50–90% of the patients developed irAEs after PD-1 or CTLA-4 blockade, and the ratio was much higher when using combined anti-PD-1 and anti-CTLA-4 therapy (2). IrAEs can occur in multiple organs, although most of them are mild to moderate. Severe irAEs, such as myocarditis, colitis, encephalitis, and pneumonitis, are life-threatening and observed in 10–20% of the patients receiving anti-PD-1 or anti-CTLA treatment.

Immune checkpoint inhibitor-related pneumonitis (CIP) is a rare but life-threatening irAE. The occurrence of CIP was initially 3–5% in a clinical trial; however, it is more commonly reported in real-world populations (3). CIP is more common in patients with lung cancer (4.1–19%) (4–6) than those with other cancer types (e.g., 10–12% in hematologic malignancy patients) (7). Severe CIP (grade 3 or higher) has been reported in as high as 4.2–11% of the patients and can lead to fatal respiratory failure with a mortality rate up to 27% (8–10). Patients with severe CIP experience temporary or permanent discontinuation of ICI therapy and may also receive glucocorticoid therapy if needed. As more ICIs have been approved as first-line therapy for a wide range of cancers, the incidence of CIP is likely to increase. However, the diagnosis and treatment of CIP is challenging because it normally presents with variable symptoms, ranging from asymptomatic to acute respiratory distress syndrome, and little is currently known about the immunopathogenesis of CIP. To improve the prognosis and reduce mortality of CIP, there is an urgent need to explore the cellular mechanism and identify therapeutic targets of CIP.

Herein, we report a clinical case of CIP in a bladder cancer patient and the application of imaging mass cytometry (IMC) techniques for the integrative analysis of the immune microenvironment in CIP.

## Case description and diagnostic assessment

**Figure 1:**
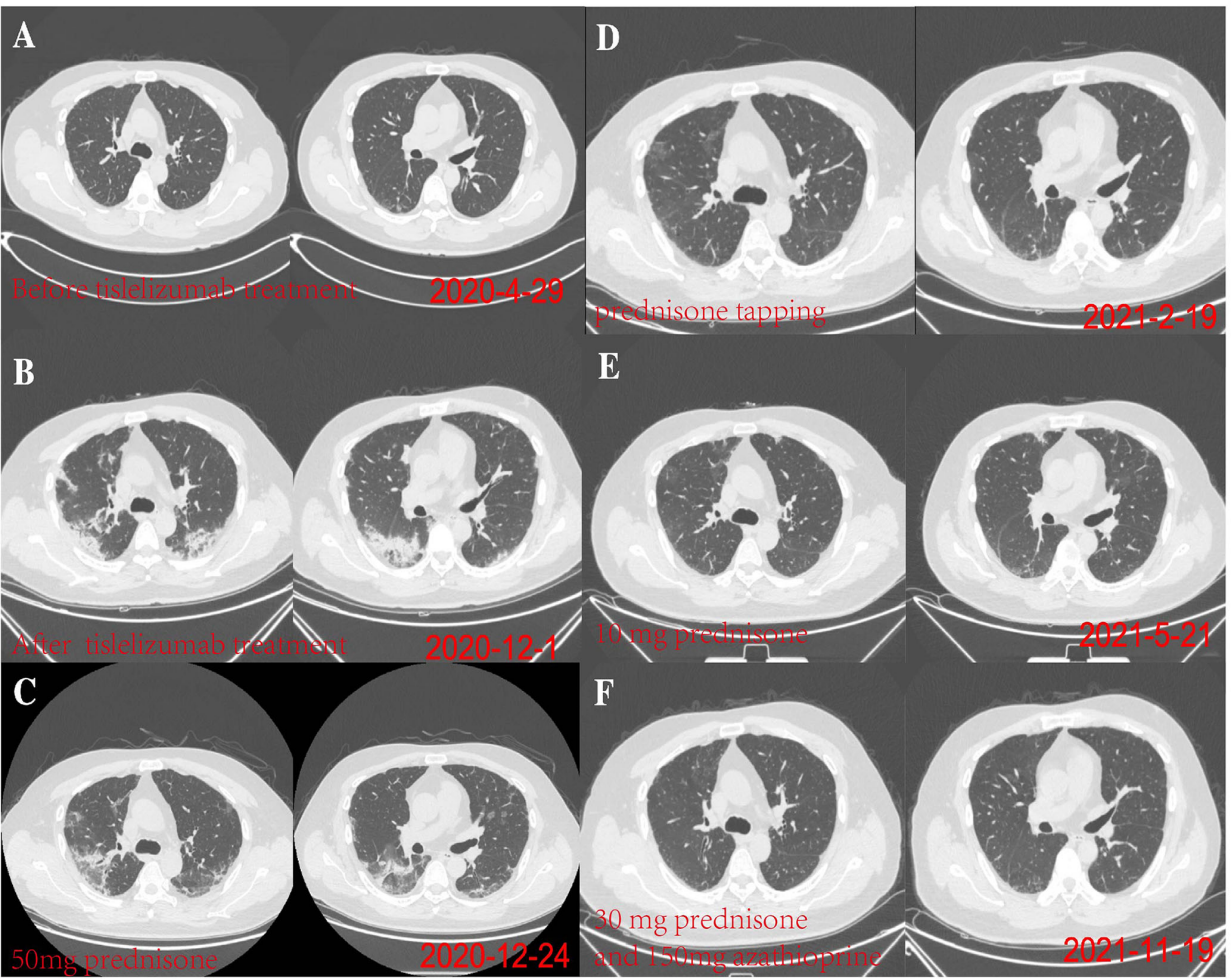
**(A)** On April 29, 2020, chest computed tomography (CT) before chemotherapy showed mild ground glass opacity in the lower lobe of the right lung. **(B)** On December 1, 2020, after 6 months of tislelizumab treatment, chest CT showed diffuse ground glass and solid opacity in both lungs, more severe in both lower lungs. **(C)** On December 24, 2020, after 3 weeks of prednisone treatment, chest CT showed pulmonary infiltration had improved. **(D)** pulmonary infiltration continued to improve on Feb 19, 2021. **(E)** Multiple subpleural ground glass lesions recurred in both lungs on May 2, 2021 (The patient received 10 mg prednisone). **(F)** The patient responded to azathioprine (150 mg per day) on November 19, 2021.

**Figure 2:**
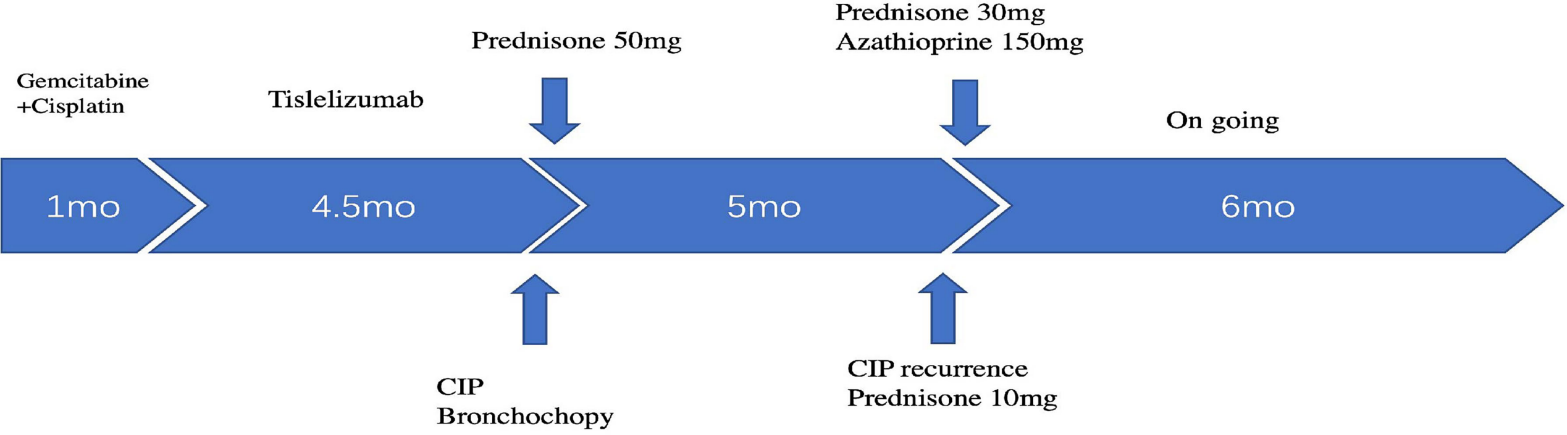
Timeline scheme of major clinical event of the patient since treatment.

**Figure 3:**
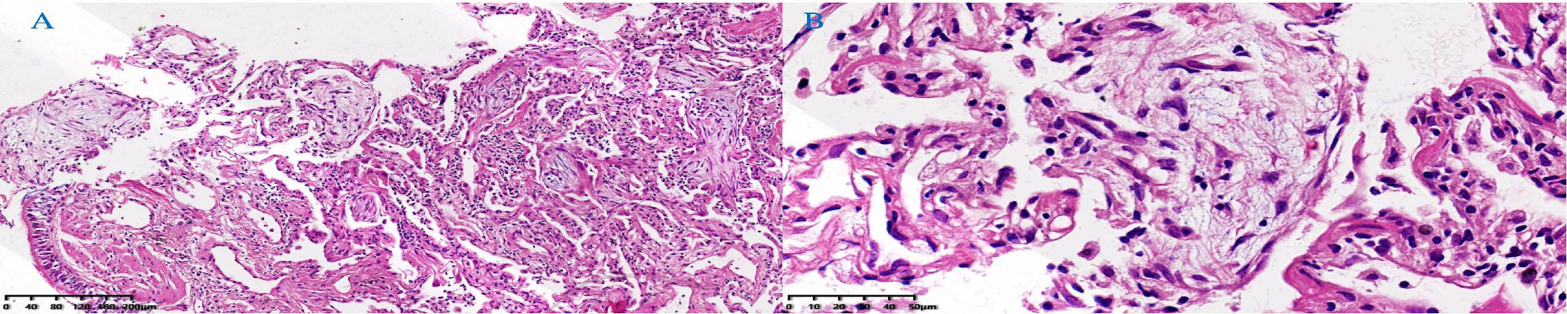
Lung tissue histopathology showed organizing pneumonia with mildly thickened alveolar septa, fibroblastic proliferation filling airspaces, and moderate lymphocytic infiltrate. **(A)** HE, low power field. **(B)** HE, high power field. (Hematoxylin-eosin, HE).

A 48-year-old male patient attended our hospital from May 2020 due to metastatic bladder cancer. The patient was a non-smoker with no history of pulmonary disease, but chest computed tomography (CT) before chemotherapy showed mild ground glass opacity in the lower lobe of the right lung (**Figure 1A**). The first cycle of chemotherapy with gemcitabine, cisplatin, was discontinued due to severe leukopenia. After that, tislelizumab was used as second-line therapy. After 6 cycles of treatment, the patient developed fever with a maximum body temperature of 37.5°C, no cough, or dyspnea (**Figure 2**). Chest computed tomography (CT) showed multiple consolidations and ground-glass lesions in both lungs (**Figure 1B**). He was transferred to the respiratory department for transbronchial cryobiopsy, and bronchoalveolar lavage fluid classification showed predominantly lymphatic cells (38%), the histopathology showed organizing pneumonia (**Figure 3**). BAL culture was negative for pathogenic bacteria, and no sign of tumor was found on the lung tissue. The patient was diagnosed with CIP according to the results of bronchoscopy,and tislelizumab was discontinued. The patient was treated with 50 mg prednisone. Chest CT showed that the ground glass and consolidation of both lungs improved significantly over 3 weeks (**Figure 1C**), and prednisone was gradually reduced. Pulmonary infiltration continued to improve on Feb 19, 2021 (**Figure 1D**). Multiple subpleural ground glass lesions recurred in both lungs on May 2, 2021 (the patient received 10 mg prednisone at that time) (**Figure 1E**). Prednisone was increased to 30 mg and 150 mg azathioprine was added. There was no sign of CIP or bladder cancer recurrence until last follow up in Nov 11, 2021 (**Figure 1F**).

**Figure 4:**
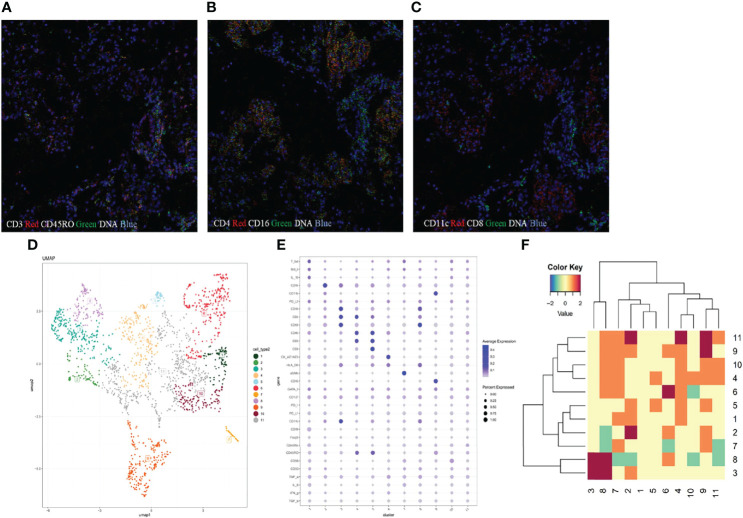
The microenvironment in patients with immune checkpoint inhibitor-related pneumonitis. **(A–C)** Imaging mass cytometry (IMC) imaging of formalin-fixed paraffin-embedded lung tissue specimen. **(D)** Statistical t-distributed stochastic neighbor embedding (tSNE) plots were generated to divide the cell populations into 11 clusters according to phenotypic similarity. **(E)** Heat map analysis of the mean expression of analyzed markers from 11 identified clusters. The expression levels are indicated by heat colors. **(F)** Neighborhood analysis revealed cell-to-cell interactions between different clusters.

Imaging mass cytometry (IMC) analysis was performed on paraffin-embedded lung parenchyma to study the microenvironment and cell-cell interactions in CIP. In brief, the major cell populations in the lung section were profiled using a 31-marker panel consisting of 22 cell surface markers and 9 intracellular markers. Lung tissues were stained with metal-tagged antibodies and multi-dimensional data were collected using Hyperion Tissue Imager (Fludigm). The details of 31 metal isotope-labeled antibodies are listed in **Table S1**. Then, the data were segmented into single cells using Cellprofiler and analyzed using the HistoCAT software (version 1.75). Thereafter, visualization of t-distributed stochastic neighbor embedding was used to generate 2-dimensional density plots, and cells were divided into 11 clusters according to the phenotypic similarity. Epithelial cells with high cytokeratin AE1/AE3 expression were included in cluster 9 and α-smooth muscle actin (ASMA) positive stromal cells (ASMA+ cells) were presented in cluster 10. The cells in clusters 1-8 represented immune cells (**Figure 4D**). We identified infiltrating immune cell types of CD14^+^ monocytes, CD16^+^ monocytes, CD4^+^ T cells, CD8^+^ T cells, and CD68^+^ macrophages in lung tissues (**Figure 4E**). Abundant T cells were present in the inflammatory region, which were mainly CD45RA^-^CD45RO^+^ CD4^+^ T cells (cluster 2) and CD45RA^-^CD45RO^+^ CD8^+^ T cells (cluster 3), indicating the infiltration of memory T cells in pneumonitis tissues (**Figure 4A**). In addition, a very small number of CD19 B cells (cluster 1) and CD56^+^ NK cells (cluster 4) were found. With regard to the myeloid-derived cells, we identified the infiltration of CD15^+^CD11b^+^ neutrophils and several monocyte subsets, including CD68^+^ macrophages, CD14^+^ monocytes, and CD11c^+^CD16^+^DC cells. CD68^+^ macrophages expressed a high level of HLA-DR (**Figure 4B**), indicating the M1-like phenotype. CD11c^+^CD16^+^ monocytes were also positive for CD4 and HLR-DR, and the data suggested that the CD4^+^ DC subset was activated. In addition, using neighborhood analysis, we found a cell-cell contact between CD8^+^ T cells and CD11c^+^ CD16^+^ DC in the microenvironment, indicating the interaction of DC-T cells and activation of CD8^+^ T cells (**Figures 4C, F**).

## Discussion

ICI therapy has caused a paradigm shift in cancer therapy. However, with the widespread use of ICI therapy, severe irAEs including CIP have attracted more attention. Previous studies reported a higher incidence and higher grade of CIP in patients with pre-existing pulmonary diseases (11). The anti-PD-1 inhibitor is known to block the interaction of tumor expressed PD-L1 and T cell expressed PD-1, thus disinhibiting T cells’ ability against tumor cells (12). However, based on its mechanism of action, it is not surprising that anti-PD-1 therapy may provoke unwanted immune activation, alter host homeostasis, and cause an inflammatory response and autoimmune disease. The physiopathology of CIP is not yet fully elucidated. The characterization of the immune profile of pneumonitis would be of value for prompting early diagnosis and intervention. In the present study, we report the case of a bladder cancer patient who received tislelizumab and was diagnosed with CIP. Using IMC analysis, we clarified the infiltration of immune cells and analyzed the microenvironment of lung tissue.

Several immune cell markers were identified as potential risk factors for CIP development. In peripheral blood, Ryosuke et al. found that the neutrophil-lymphocyte ratio was significantly elevated 4 weeks before symptoms appeared in CIP patients and could serve as a significant prognostic factor (13). In addition, increased levels of IL-6, IL-10, PLR, LDH, and decreased levels of ALC, and ALB were found in the CIP serum. Previous work has also identified dysregulated and lymphocyte-predominant alveolar immune profiles in bronchoalveolar lavage fluid; they found the enrichment of proinflammatory T cells, Th17/Th1 and central memory (CD62L^hi^CD45RA^low^) T cells, decreased Treg cells, as well as the upregulation of proinflammatory cytokines (such as TNFα and IL-6) in CIP patients (14–16). However, studies on lung tissues are limited. The imaging of the lung section might provide more detailed information about the immune cell distribution and infiltration during inflammation.

IMC is a mass spectrometer-based multiparametric technology and allows for the simultaneous detection of multiple (up to 40) protein markers at a single cell level using heavy metal-tagged antibodies. The multiplexed analysis provides an integrative information of tissue microenvironment, and generates high dimensional data with formalin-fixed paraffin-embedding or snap-frozen tissues. The use of metals, although results in lower resolution (1 μm) than fluorescence imaging, decreases the interference of spectral overlap effect and is attractive for strongly autofluorescence tissue detection. In addition, IMC preserves complex tissue contexture and provides *in situ* characterization of spatial interaction between immune cells (17). It had great potential of identifying irAEs related immune cell subsets within the lung sections. However, it also faces several challenges, for example, there is currently no standard for the validation of antibody performance in IMC panel and there is no signal amplification of secondary antibody in IMC system, it is thus necessary to optimize the detection protocol for low-expressed markers (18).This method has recently been used to characterize the immune cells in lung squamous cell carcinoma and has identified a large number of infiltrating immune cells, including T cells, B cells, and myeloid-derived suppressor cells (19). In the current study, we used 31 antibodies targeting markers for cell lineage, activation, and memory subsets. We observed the infiltration of CD4 and CD8 T cells, but not B cells or NK cells. We also identified the pattern of memory T cells. The finding suggested that abnormal infiltration of memory T cells may have played a role in this patient’s CIP, which was consistent with the findings in other irAEs (e.g., myocarditis) (20, 21). In addition, we found the accumulation of CD4^+^HLR-DR^+^DC and DC-CD8^+^T interaction in inflammatory tissues, CD4^+^DC represents a subset of DC that more efficiently stimulated Th1 and Th2 response (22), the data suggested the activation of memory T cells in this CIP patient.

This patient developed CIP after receiving tislelizumab treatment. Glucocorticoid treatment was effective, but CIP relapsed during the dose reduction process. Asher et al. reported This unique event of “pneumonitis flare” speculates that there may be some undiscovered respiratory factors, or due to the long half-life of anti-PD-1 agents (23). It is recommended to longer time of immunosuppressive therapy for these patients. We truly hope more extensive IMC analysis of biopsy samples in CIP especially before and after the biopsy that optimal therapeutic options will be identified.

## Conclusion

The case demonstrated the infiltration of immune cell profile (including memory CD4^+^T and CD8^+^T cells, CD4^+^DCs) in the CIP and suggested a potential application of IMC for the diagnosis and understanding the immune cell mechanism of irAEs. Further investigation is warranted to explore the immune patterns specific to CIP by comparison of sensitivity to glucocorticoid therapy.

## Data Availability Statement

The raw data supporting the conclusions of this article will be made available by the authors, without undue reservation.

## Ethics Statement

The studies involving human participants were reviewed and approved by the ethics committee of Peking University First Hospital. The patients/participants provided their written informed consent to participate in this study. Written informed consent was obtained from individuals for the publication of any potentially identifiable images or data included in this article.

## Author Contributions

Conception/Design: YC. Provision of study material or patients: YC, XZ, KS, MZ, HW, YX, and GW. Collection and/or assembly of data: YC, QH, and X-MW. Data analysis and interpretation: QH and X-MW. Manuscript writing: YC and QH. All authors contributed to the article and approved the submitted version.

## Conflict of Interest

The authors declare that the research was conducted in the absence of any commercial or financial relationships that could be construed as a potential conflict of interest.

The reviewer BZ declared a shared affiliation with the author X-MW to the handling editor at time of review.

## Publisher’s Note

All claims expressed in this article are solely those of the authors and do not necessarily represent those of their affiliated organizations, or those of the publisher, the editors and the reviewers. Any product that may be evaluated in this article, or claim that may be made by its manufacturer, is not guaranteed or endorsed by the publisher.
